# rANOMALY: AmplicoN wOrkflow for Microbial community AnaLYsis

**DOI:** 10.12688/f1000research.27268.1

**Published:** 2021-01-07

**Authors:** Sebastien Theil, Etienne Rifa

**Affiliations:** 1 Université Clermont Auvergne, INRAE, VetAgro Sup, UMRF, F-15000, Aurillac, France

**Keywords:** 16S, ITS, amplicon sequencing, metagenomics, microbial community, R package

## Abstract

Bioinformatic tools for marker gene sequencing data analysis are continuously and rapidly evolving, thus integrating most recent techniques and tools is challenging. We present an R package for data analysis of 16S and ITS amplicons based sequencing. This workflow is based on several R functions and performs automatic treatments from fastq sequence files to diversity and differential analysis with statistical validation. The main purpose of this package is to automate bioinformatic analysis, ensure reproducibility between projects, and to be flexible enough to quickly integrate new bioinformatic tools or statistical methods. rANOMALY is an easy to install and customizable R package, that uses amplicon sequence variants (ASV) level for microbial community characterization. It integrates all assets of the latest bioinformatics methods, such as better sequence tracking, decontamination from control samples, use of multiple reference databases for taxonomic annotation, all main ecological analysis for which we propose advanced statistical tests, and a cross-validated differential analysis by four different methods. Our package produces ready to publish figures, and all of its outputs are made to be integrated in Rmarkdown code to produce automated reports.

## Introduction

Studies of microbial communities tends to become a daily routine analysis for lots of laboratories and the main method to explore microbial diversity is metabarcoding, which is an amplicon targeted sequencing method (16S for bacteria and ITS for fungi). Metabarcoding generates a large amount of data and a lot of applications already exist for their processing (FROGS ^1^, qiime ^2^). Methods and software are continuously evolving and the main challenge for bioinformaticians is to implement the most recent and effective ones in their analysis. Here we present rANOMALY, a scalable and lightweight R package which is able to handle every step of a metabarcoding analysis, from read cleaning, contaminant filtering, taxonomic assignment, to advanced statistical analysis. rANOMALY is fully implemented in R language in which each step correspond to one function, allowing to easy implementation of new features or tools while being easy to use and maintain. The package allows the workflow to be executed on any R environment. rANOMALY only needs a CSV table describing the metadata for each sample, and a folder containing the corresponding fastq files as input. It can produce high quality figures for Rmarkdown reports along with statistical tests ready for publication.

**Figure 1.**
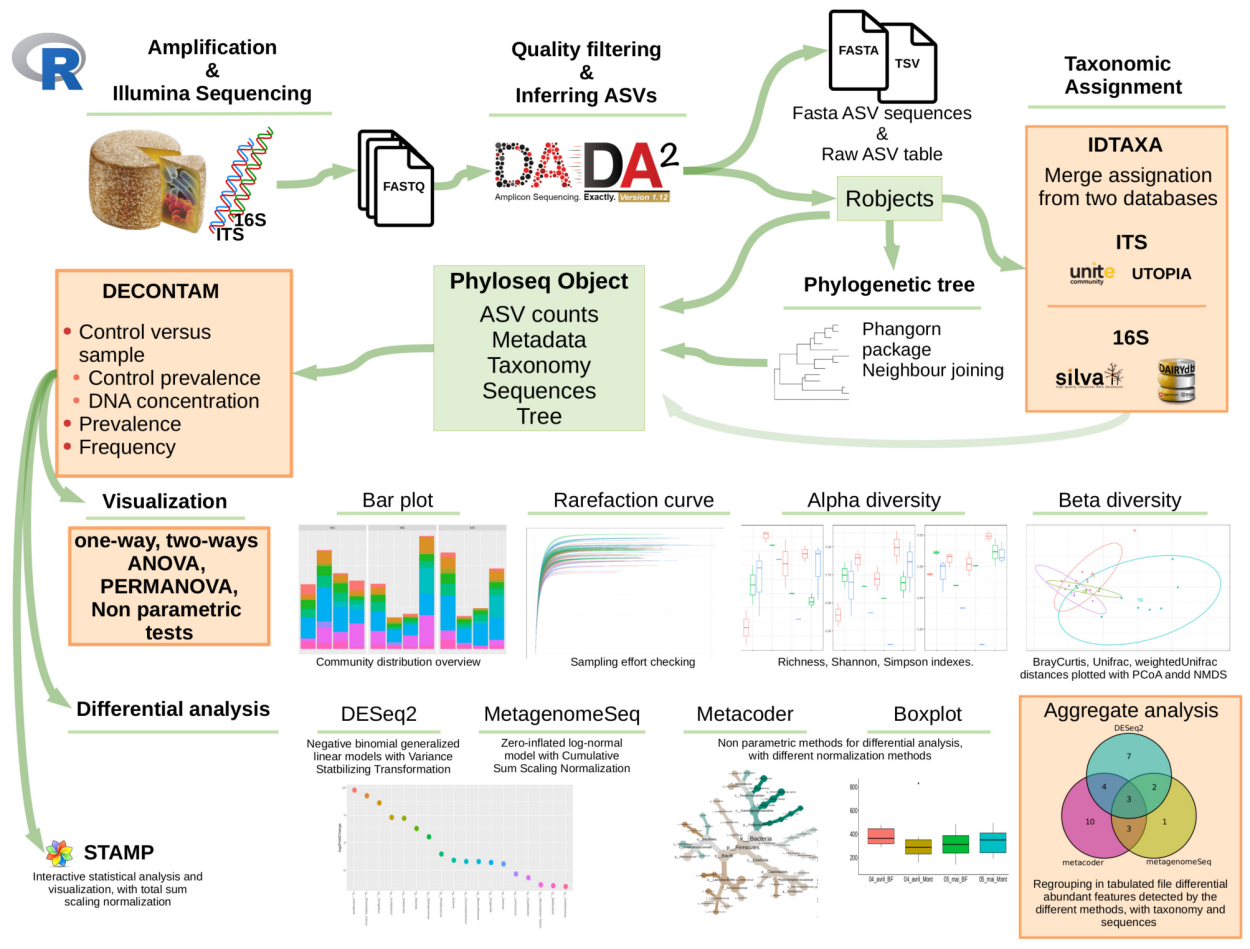
rANOMALY workflow.

The workflow is illustrated in Figure 1.

## Implementation

rANOMALY is an R package depending on other CRAN, Bioconductor, and git R packages. It is easy to install via devtools::install_git function.

### Raw sequence processing

Samples must have been previously demultiplexed into one file per sample with the file name following this syntax: {sampleid}_R[12].fastq. The denoising process is handled using the dada2 R package ^3^ which produces amplicon sequence variants (ASV) as a taxonomic unit. This improves resolution of the potential presence of microbial organisms by using a prediction model to correct sequencing errors before aggregating similar sequences. rANOMALY handles processing any region of 16S (V1 to V9) and ITS amplicon (ITS1, ITS2) sequences, in which an additional step of cutadapt ^4^ removes ITS probes left in some short sequences. For 16S amplicon, primers are trimmed based on the primer sequence length. ASV identifiers are sequences translated into MD5 hashes which are unique identifiers based on the DNA sequences offering the possibility to be compared between projects. This step results in an object with representing sequences, a raw ASV counts table and a text file containing statistics from the denoising process.

### Taxonomic assignment

Taxonomic assignments of ASVs are carried out by IDTAXA (part of the DECIPHER package), an algorithm based on a machine learning method ^5^. We implemented a functionality to compute assignment with two reference databases. For example, 16S amplicon sequences can be assigned with an environment specific database (DAIRYdb ^6^, HITdb ^7^, MIDAS ^8^) and a general database like SILVA ^9^ or GreenGenes ^10^. The assignment with the best confidence and the lowest rank is kept, thus increasing assignment depth and accuracy. As taxonomy can differ between reference databases, we implemented a taxonomy validation step in rANOMALY to unravel taxonomy inconsistency like taxa with multiple ancestors or empty ranks. As supplementary features, rANOMALY functions allow users to create their own IDTAXA formatted specific reference database. The first step consists of filling the taxonomic table empty fields with the last known rank, and checking for taxonomy incongruencies as in the assignment function. A taxid file as used with RDP classifier ^11^ is constructed and then a last function takes as input, the corrected taxonomy table, the fasta file and the taxid file to generate the IDTAXA formatted database.

### Phylogenetic tree

Phylogenetic tree is generated in three steps. First, sequences are aligned with AlignSeqs function from DECIPHER package ^12^ by the guided-tree method. Then, distance matrix is calculated with the dist.ml function from the phangorn package. And finally, neighbour joining and pml function computes the likelihood of the phylogenetic tree.

The abundance, metadata, taxonomic table, reference sequences and phylogenetic tree are merged into a phyloseq object ^13^.

### Decontamination

ASVs have the advantage of enabling the distinction between contaminants and the real community. We have integrated the decontam package ^14^ into the workflow. Indeed using OTU based clustering methods can agglomerate contaminant sequences with real sample sequences, the whole cluster could hence be considered as contaminant by mistake. Working with ASVs allows the use of R package decontam which will sensitively exclude contaminant ASVs. It integrates two main methods, one based on the prevalence of the contaminant in the control samples, and another one based on the DNA concentration of the samples. Moreover, our decontamination step allows users to apply various filters such as low ASVs frequency, low ASVs prevalence in real samples, and the minimum number of reads per sample.

### Graphical plots and statistical analyses

Statistical analyses are key features of rANOMALY workflow. Main descriptive analyses are integrated thanks to phyloseq functionalities. In addition, we automatized graphical representations and advanced statistical tests for alpha, beta diversity and composition plots. Above all, we have included the four most up-to-date differential analyses to assess differentially abundant features.

### Community composition plots, alpha and beta diversity analyses

rANOMALY allows users to explore microbial community composition with three different types of plots : classical interactive bar plot ^15^ of raw and relative taxa abundances, rarefaction curves to check sampling effort, and Krona interactive pie charts ^16^. 

**Alpha diversity** indices representing the specific richness are calculated (Richness, Simpson, Shannon...) with the vegan R package ^17^. We added statistical tests such as multi-factors analysis of variance and pairwise Wilcoxon tests to assess significant differences between tested categories. We included repeated measures ANOVA to handle within-subjects variation. For example, it can be used when there are measures taken on the same individuals at different time points. This step outputs graphical representations and tables with results of statistical tests which are both saved in files and returned as list objects for markdown reports.

**Beta diversity** analysis allows users to estimate the community differences between two samples, it is also based on the vegan package ^17^. rANOMALY can calculate all different distances such as the one based on ASVs abundance (BrayCurtis), rank based Jaccard indices, and phylogenetic distances as UniFrac, weighted Unifrac. Graphical representations PCoA, NMDS and more are available. This analysis can be processed at different taxonomic levels and categorical factors chosen by the user. The additional statistical test of PERMANOVA uses the distance matrix to determine if microbial communities of sample groups are significantly different from each other. We added a pairwise PERMANOVA ^18^ test to confirm significant differences between specific group of samples.

### Differential analysis

Differential analyses are meant to assess potential differentially abundant taxas between tested conditions chosen by users. rANOMALY wraps three methods:

- DESeq2 ^19^ uses negative binomial generalized linear model with a variance stabilizing transformation on abundances.
- metagenomeSeq ^20^ uses a zero-inflated log-normal model with cumulative sum scaling normalization.
- metacoder ^21^ applies a total sample sum normalisation and uses a non-parametric Wilcoxon Rank Sum test to compare the log ratio of mean proportions.

The use of multiple methods for differential analysis allows the user to investigate which feature can be considered as differentially abundant between conditions. rANOMALY function using DESeq2 and metagenomeSeq outputs tables and plots with significant features. Metacoder outputs heat tree plots allowing users to infer differentially abundant features at each taxonomy rank and their position on the phylogenetic tree. With basic data management, and according to the identification of all significant differentially features (i.e. ASVs, genus, species) from the three methods, we generate a single table to find significant features in one or more methods and ease the interpretation. Additional information are added to the final table, like mean relative abundance for each feature and condition. A column in which condition features are significantly more abundant, features taxonomies and related sequences are added to the aggregated table. To complete differential analysis, we have included the well recognized PLS-DA from the mixOmics package ^22^. It is a supervised classification method allowing users to identify features discriminating the sample groups.

### Additional features and export

Functions and procedures are available to help the user to generate additional figures or to export the data to third party software. For instance, shared taxa between conditions are useful to explore. We use a function that can generate Venn diagrams, or for more complex visualisation, we can produce files readable by Cytoscape ^23^ to produce shared taxa networks. Krona diagrams can be displayed to explore sample microbial composition, where samples can be merged by a specified factor. rANOMALY allows users to generate inputs for STAMP ^24^, which is a graphical software that provides statistical hypothesis tests and exploratory plots for analysing taxonomic profiles.

### Operation

rANOMALY requires R 3.6.3 or upper and can be run on any operating system with common specifications (1Go disk space, 4Go RAM, multicore CPU is recommended).

## Use case

For this example we are using a dataset from Fretin *et al.* study ^25^ in which samples are from four different environments: cow milk, cow cheese (rind and core) and cow teat skin. This dataset and metadata are available on NCBI-SRA website: BioProject accession PRJNA421256. To ease access to this dataset, fastq files along with pre-formatted metadata are available on this repository. 

### Install

Up to date code is hosted by the INRAE gitlab, users can simply download and install the package in R console with following command lines.

# Install ranomaly
install.packages("devtools")
devtools::install_git("https://forgemia.inra.fr/umrf/ranomaly")

# Call ranomaly package
library(ranomaly)
setwd("PATHTOWORKINGDIRECTORY")

# Create the analysis directory
dir.create("./analysis")

### Processing of raw sequences

***ASV definition with DADA2*.** The first step is to define ASVs thanks to the dada2 package. In rANOMALY, only one function is needed to compute all the different steps require from this package. Here sample names are extracted from the file name, thus be sure that files name match samples name in the metadata file.

# ASV resolution step, raw ASV table and representative sequence generation
dada_res = dada2_fun(amplicon = "16S", path = "./fastq_files", outpath = "./analysis/01_results_dada/",
    plot = FALSE, compress = FALSE, verbose = 1, paired = FALSE, torrent_single = FALSE,
    returnval = TRUE)

Main outputs of this function are:

- read_tracking.csv summarizes the read number after each filtering step ( Table 1).
- raw_otu-table.csv the raw ASV table.
- rep-seqs.fna fasta file with all representative sequences for each ASV.
- robjects.Rdata with saved dada_res list containing raw ASV table and representative sequences in objects otu.table, seqtab.export, seqtab.nochim.

**Table 1.** Read tracking table overview.

sample.id	input	ﬁltered	denoisedF	nonchim
SRR6365127.fastq	35690	33511	33402	33402
SRR6365128.fastq	38871	38183	38111	38046
SRR6365129.fastq	50970	49765	49631	49092
SRR6365130.fastq	44207	42657	42524	42524
SRR6365131.fastq	68008	66517	66257	66087
SRR6365132.fastq	44014	42158	41945	41848
SRR6365133.fastq	36704	35884	35799	35444
...	...	...	...	...

# Adjust sample names to match with metadata
colnames(dada_res$otu.table) = tools::file_path_sans_ext(colnames(dada_res$otu.table))

***Taxonomic assignment*.** assign_taxo_fun function uses IDTAXA function from DECIPHER package, and allows to use two different databases. It keeps the best assignment on two criteria, resolution (depth in taxonomy assignment) and confidence (value givenby IDTAXA). The final taxonomy is validated by checking for multiple ancestors ( *i.e.* same species assigned to different genus) and incongruities ( *i.e.* empty fields or incomplete lineage) correction step.

We share the latest databases we use in the IDTAXA format in this link. Users can also generate your own IDTAXA formatted database following those instructions and scripts we provide at this page.

# Taxonomy assignment step
tax.table = assign_taxo_fun(dada_res = dada_res, id_db = c("PATHTO/SILVA_SSU_r138_2019.RData",
    "PATHTO/DAIRYdb_v1.2.4_20200604_IDTAXA.rdata"), verbose = 3, output = "./analysis/02_idtaxa_silva_ddb")

Main file outputs:

- robjects.Rdata with taxonomy in phyloseq format in tax.table object.
- final_tax_table.csv the final assignation table ( tax.table) outputed in CSV format.
- allDB_tax_table.csv raw assignations from the two databases, mainly for debugging.

***Phylogenetic tree*.** The phylogenetic tree from the representative sequences is generated using phangorn and DECIPHER packages.

tree = generate_tree_fun(dada_res)

***Phyloseq object*.** To create a phyloseq object, we need to merge four objects and one file:

- the ASV table otu.table and the representative sequences seqtab.nochim in dada_res variable.
- a taxonomy table ( tax.table).
- the phylogenetic tree ( tree).
- metadata from from csv file.

# Phyloseq object generation
data = generate_phyloseq_fun(dada_res = dada_res, tax.table = tax.table, tree = tree,
    metadata = "./sample-metadata.csv", output = "./analysis/03_phyloseq/")

***Decontamination*.** The decontam_fun function uses decontam R package along with control samples (PCR control) to filter out contaminants. The decontam package offers two main methods, frequency and prevalence (users can also combine those methods). For frequency method, it is mandatory to have the DNA concentration of each sample in the phyloseq object (and hence in the metadata.csv). The prevalence method does not need DNA quantification, this method allows to compare presence/absence of ASV between real samples and control samples and then identify contaminants.

**Tips:** sequencing plateforms often quantify the DNA before sequencing, but do not usually give the information. Just ask for it ;).

Our function integrates the basic ASV frequency $\left(Freq=\frac{nb\_reads\_ASV}{nb\_total\_reads}\right)$ and minimum prevalence in overall samples filtering. We have also included an option to filter out ASV based on their taxa names for known laboratory recurrent contaminants.

Main outputs:

- robjects.Rdata with contaminant filtered phyloseq object named data.
- Exclu_out.csv list of filtered ASVs for each filtering step.
- Krona plot before and after filtering.
- raw_asv-table.csv and relative_asv-table.csv.
- venndiag_filtering.png venn diagram showing the repartition of filtered ASVs by decontamination methods.

Here we are going to filter out ASVs representing less than 0.1% ( freq) of the reads and that are present in more than 4 samples ( prev). Moreover, we are excluding "unassigned" taxas for this use case. Our sample dataset do not contain control samples, this step will be skipped.

data_filtered = decontam_fun(data = data, domain = "Bacteria", skip = TRUE, number = 4000,
    freq = 0.001, prev = 4, unassigned = TRUE, krona = TRUE, output = "./analysis/04_decontam/")

We obtain the final phyloseq object used for downstream analysis:

> data_filtered
phyloseq-class experiment-level object
otu_table()   OTU Table:         [ 59 taxa and 48 samples ]
sample_data() Sample Data:       [ 48 samples by 34 sample variables ]
tax_table()   Taxonomy Table:    [ 59 taxa by 7 taxonomic ranks ]
phy_tree()    Phylogenetic Tree: [ 59 tips and 57 internal nodes ]
refseq()      DNAStringSet:      [ 59 reference sequences ]

### Plots, diversity and statistic analyses

**Figure 2.**
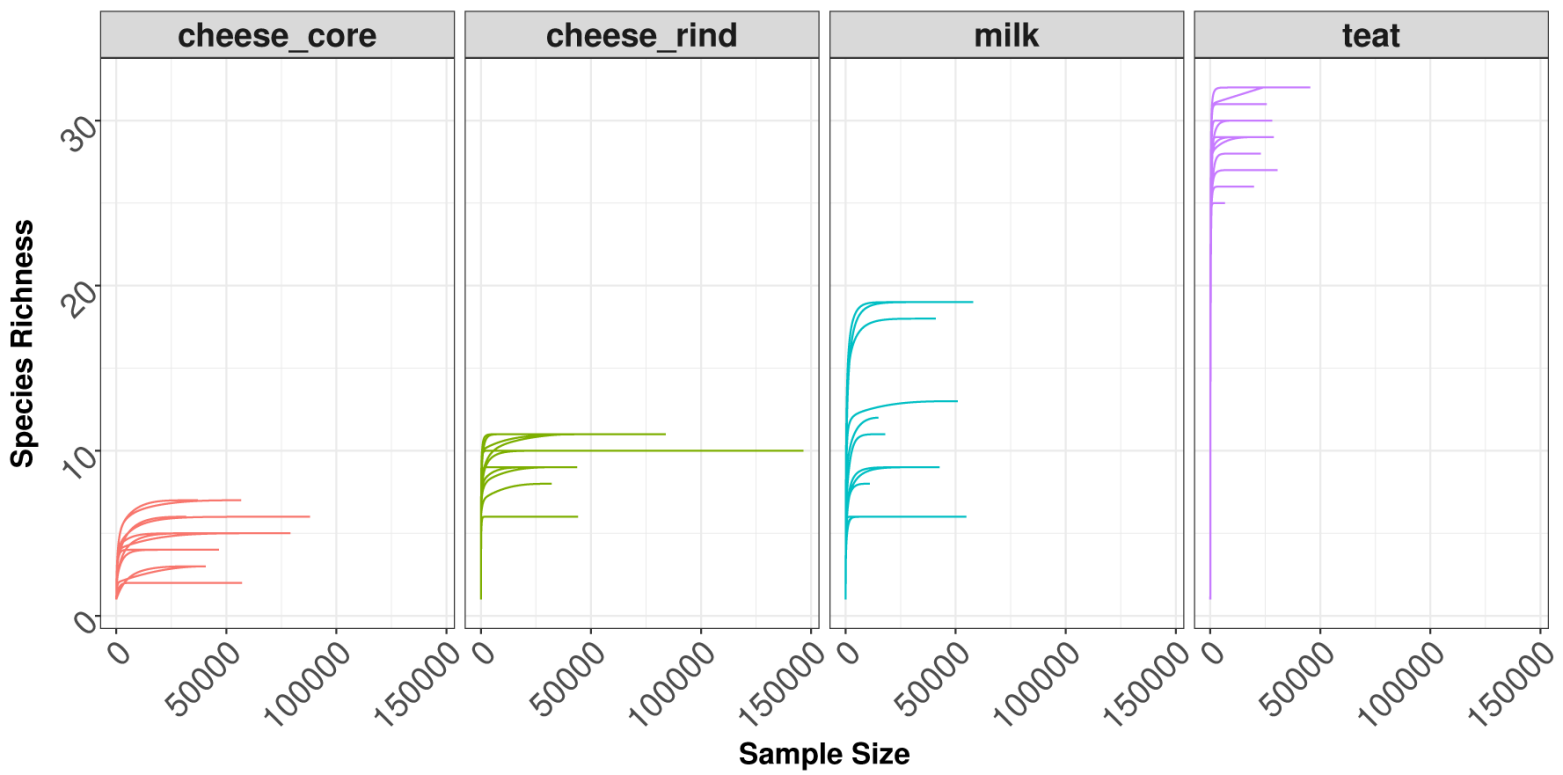
Rarefaction plot for all samples, separated by source_location factor.

***Rarefaction curves*.** In order to observe the sampling depth of each sample we start by plotting rarefaction curves. Those plots are generated by plotly which makes them interactive. ( Figure 2)


rareplot= rarefaction(data_filtered,"source_location", 
                        100)


**Figure 3.**
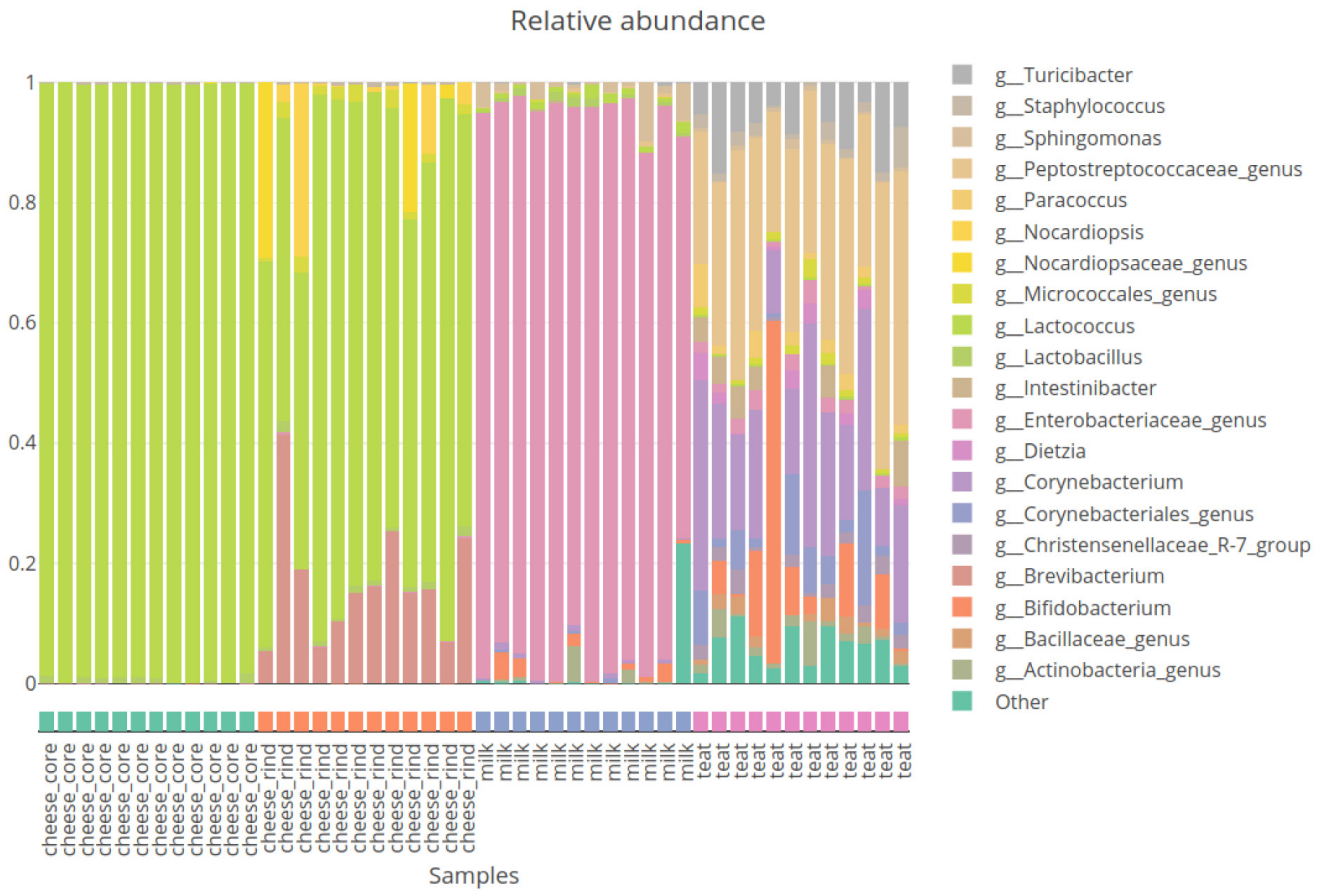
Composition plot of relative abundance of 20 most abundant genus.

***Community composition plot*.** The bars_fun function allows user to generate interactive community composition plot. Figure 3 presents the composition plot with relative abundances for the top 20 genera existing in our samples. The function allows to plot at different taxonomy rank and to modify the number of taxa to show.

Here two arguments controlling the composition plot aesthetics:

- Ord1 option order the sample along the X axis.
- Fact1 option control labels of the X axis. Fact1="sample.id" if user don’t want the sample to be renamed.

source_location factor shows very different bacterial community between milk, cheese and cow teats environments.

# Relative abondance
prel = bars_fun(data_filtered, "Genus", top = 20, Ord1 = "source_location", Fact1 = "source_location",
    relative = TRUE, outfile = "plot_compo_rel.html")

**Figure 4.**
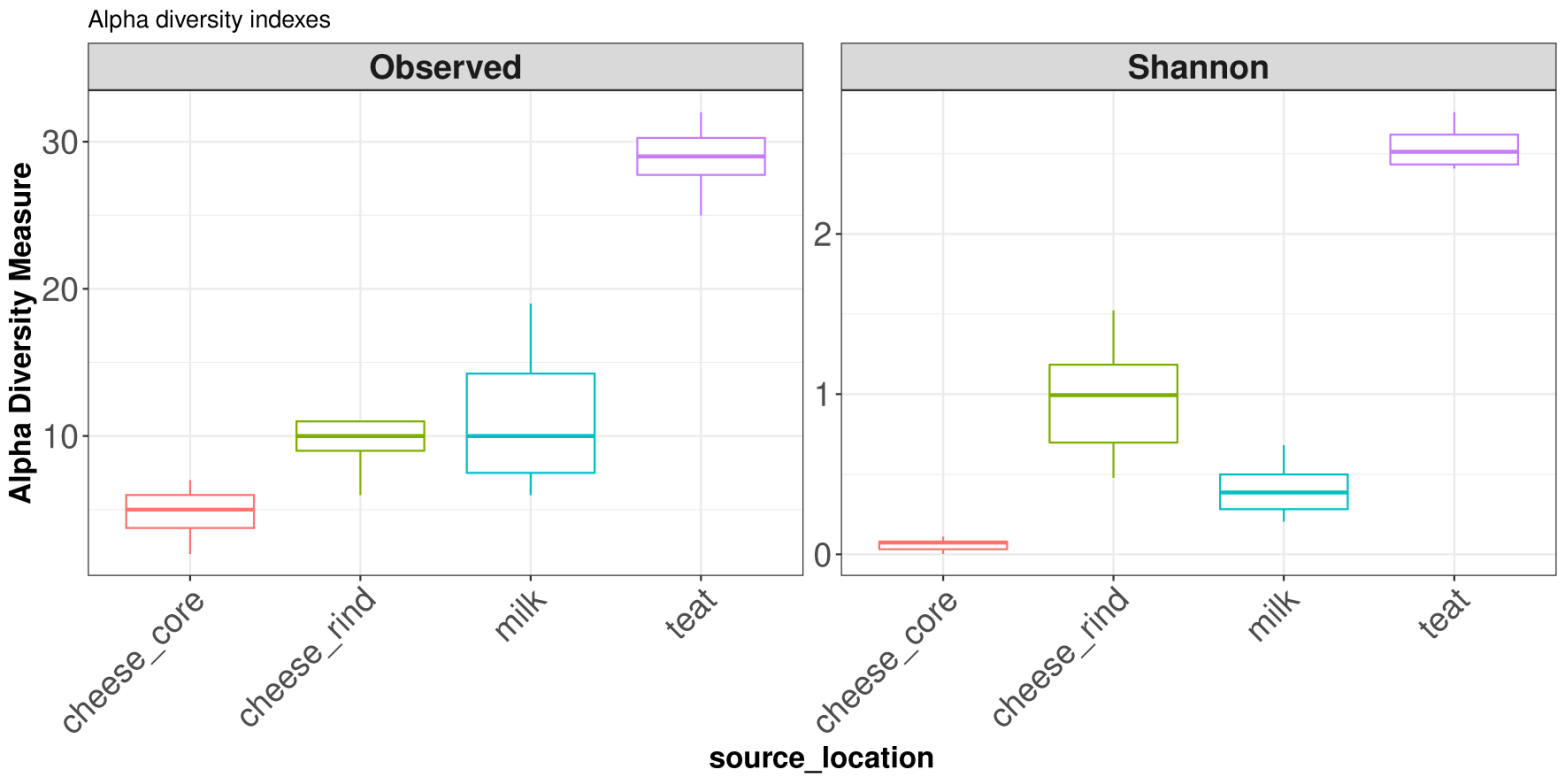
Boxplot of diversity alpha indexes.

***Alpha diversity*.** The alpha_diversity_fun function can computes various alpha diversity indexes. It uses the estimate_richness function from phyloseq (Available measures : Observed, Chao1, ACE, Shannon, Simpson, InvSimpson, Fisher). Here we calculate ASV richness and Shannon index and carry out an analysis of variance on the source_location factor. Sequencing depth is automatically taken into account in this test. A pairwise wilcoxon test is added to ANOVA to define which group might be significantly different from others. Figure 4 shows boxplots of diversity indices, cow teats environment has much more ASV than other environments. Shannon index reveals more differences between cheese rinds and cheese cores. Cheese rinds show a higher Shannon index highlighting a more balanced bacterial community.

divAlpha = diversity_alpha_fun(data = data_filtered, output = "./analysis/07_all_res/plot_div_alpha/",
     column1 = "source_location", measures = c("Observed", "Shannon"))

Results of the analysis of variance and pairwise wilcoxon test on Shannon index:

# > divAlpha$Shannon
# $anova
#                  Df Sum Sq Mean Sq F value  Pr(>F)
# Depth             1   0.83    0.83   9.203 0.00409 **
# source_location  3  98.37   32.79 365.661 < 2e-16 ***
# Residuals        43   3.86    0.09
# ---
# Signif. codes:  0 ‘***’ 0.001 ‘**’ 0.01 ‘*’ 0.05 ‘.’ 0.1 ‘ ’ 1
#
# $wilcox_col1
#           milk past_rind raw_rind
# past_rind    0        NA       NA
# raw_rind     0         0       NA
# teat         0         0        0

The alpha_diversity_fun function returns a list which contains:

- boxplots comparing conditions with chosen indices. ( $plot)
- a table of indices values. ( $alphatable)

And for each of the computed indices :

- an ANOVA analysis. ( ${measure}$anova)
- a pairwise wilcox test result comparing conditions and giving the pvalue of each comparison tested: ${measure}$wilcox_col1: wilcox test results on the first or unique factor, ${measure}$wilcox_col2_fdr: wilcox test results on the second factor, ${measure}$wilcox_col2_collapsed: wilcox test results on collapsed factor 1 and factor 2.
- a mixture model if your dataset includes repeated measures, ie. column3 option. ( ${measure}$anovarepeat, ${measure}$mixedeffect)

***Beta diversity*.** The diversity_beta_light function allows to generate specific tests and figures ready to publish in rmarkdown report as in the example below. It is based on the vegan package function vegdist for the distance calculation and phyloseq-extended in addition to ordinate funtion for the ordination plot.

We include statistical tests to ease the interpretation of results. A permutational ANOVA is carried out on matrix distance to compare groups by testing if centroids and dispersion are equivalent for all groups. User have to inform col argument and optionally cov (covariable) to assess PERMANOVA to determine significant differences between groups. A pairwise-PERMANOVA is processed to determine which condition is significantly different from another (based on p-value). 

As a return, you will get a list that contains:

- An ordination plot ( $plot).
- The permANOVA results ( $permanova).
- The pairwise permANOVA ( $pairwisepermanova)

**Figure 5.**
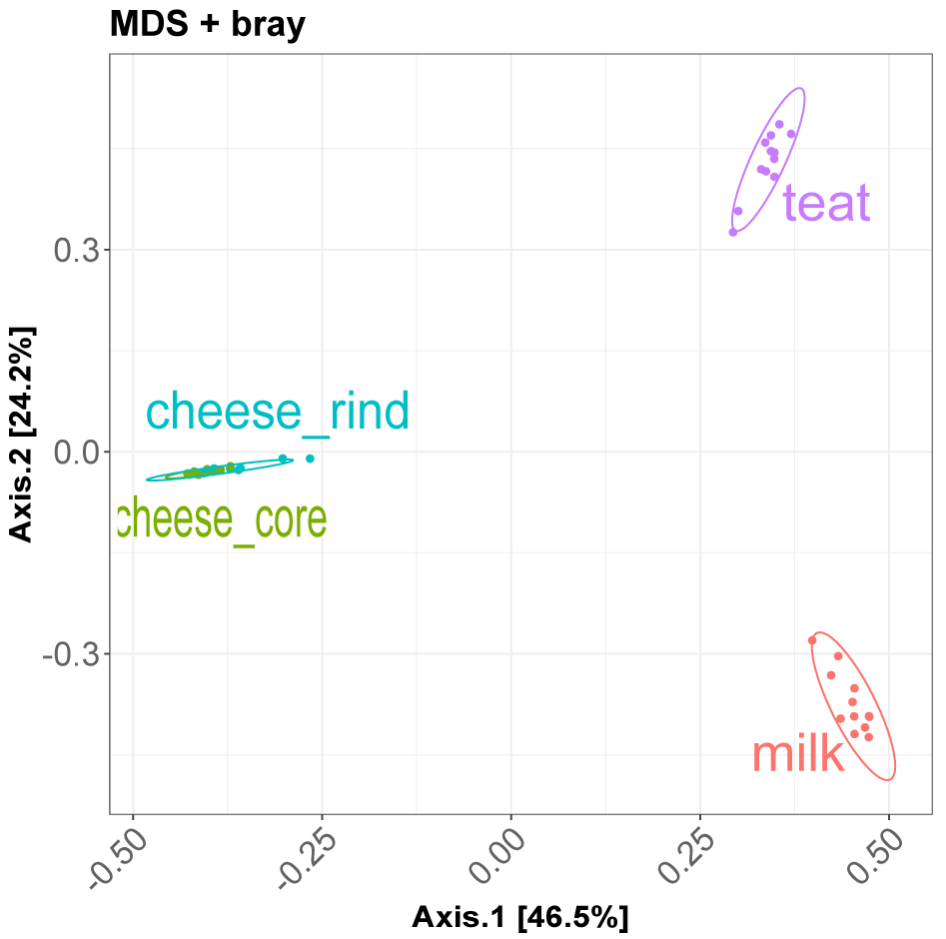
Ordination plot: MDS on Braycurtis distance.

Here we present results of beta diversity analysis on source_location factor, Figure 5 show ordination plot and it confirms the big differences between community of cheese, milk and cow teats.

divBeta = diversity_beta_light(psobj = data_filtered, output = "./analysis/07_all_res/plot_div_beta/",
     col = "source_location", cov = NULL, dist0 = "bray", ord0 = "MDS")

The permanova tests on BrayCurtis distance shows significant p-value for the source_location factor. Pairwise permanova test is used to define which level of the factor tested is significantly different from others.

divBeta$permanova
# Permutation: free
# Number of permutations: 1000
#
# Terms added sequentially (first to last)
#
#                 Df SumsOfSqs MeanSqs F.Model      R2   Pr(>F)
# Depth            1    2.4044  2.4044  27.072 0.14822 0.000999 ***
# source_location  3    9.9981  3.3327  37.523 0.61634 0.000999 ***
# Residuals       43    3.8191  0.0888         0.23544
# Total           47   16.2217                 1.00000
# ---
# Signif. codes:  0 ‘***’ 0.001 ‘**’ 0.01 ‘*’ 0.05 ‘.’ 0.1 ‘ ’ 1

divBeta$pairwisepermanova
#                        pairs Df SumsOfSqs   F.Model        R2 p.value p.adjusted sig
# 1        milk vs cheese_core  1 5.2215038 85.356493 0.7950753   0.001     0.0012   *
# 2        milk vs cheese_rind  1 4.8836384 53.310404 0.7078757   0.001     0.0012   *
# 3               milk vs teat  1 3.9377389 26.676358 0.5480352   0.001     0.0012   *
# 4 cheese_core vs cheese_rind  1 0.2640387  4.573476 0.1721068   0.008     0.0080   *
# 5        cheese_core vs teat  1 4.7206284 41.504931 0.6535702   0.001     0.0012   *
# 6        cheese_rind vs teat  1 4.3806123 30.384779 0.5800307   0.001     0.0012   *

***Differential analysis*.** We choose three different methods to process differential analysis which is a key step of the workflow. The main advantage of the use of multiple methods is to cross validate deferentially abundant taxa between tested conditions. For this use case, we choose to focus on milk and cow teat environment to compare community at genus level.

**Metacoder** Metacoder is the most simple differential analysis tool of the three. Counts are normalized by total sum scaling to minimize the sample sequencing depth effect and it uses a Kruskal-Wallis test to determine significant differences between groups. The metacoder_fun function allows the user to choose the taxonomic rank, which factor to the test ( column1), and a specific pairwise comparison ( comp) to launch the differential analysis.

**Figure 6.**
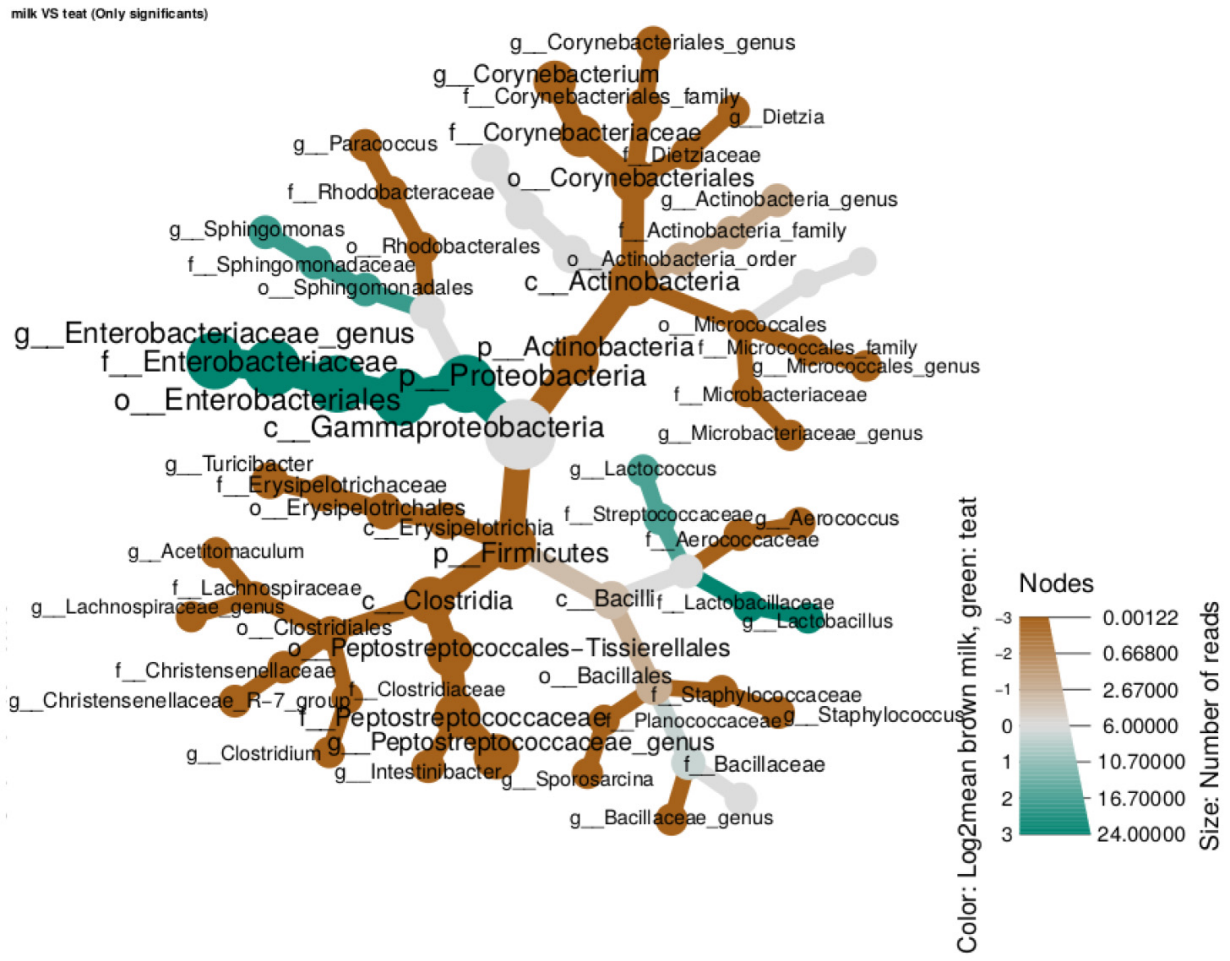
Heattree of significant features generated by metacoder package.

It produces pretty graphical trees, representing taxas present in both groups and coloring branches depending on which group this taxa is more abundant ( Figure 6). Two trees are produced, a raw one, where everything is displayed and a filtered one where only significant features are represented (p-value <= 0.05).

It produces pretty graphical trees, representing taxas present in both groups and coloring branches depending on which group this taxa is more abundant (Figure 6). Two trees are produced, a raw one, where everything is displayed and a filtered one where only significant features are represented (p-value <= 0.05).

# Metacoder
mtc = metacoder_fun(data = data_filtered, output = "./analysis/07_all_res/DA/metacoder",
    column1 = "source_location", rank = "Genus", signif = TRUE, plottrees = TRUE,
    min = "10", comp = "milk^~^teat")

Main output is a list with :

- for each comparison ( ${comparison}), two heattree, one with all features ( ${comparison}$raw) and an other one with only significant features ( ${comparison}$signif).
- a table with all wilcoxon test results ( $table).

**DESeq2** DESeq2 is a widely used method, primarily for RNAseq applications, for assessing differentially expressed genes between controlled conditions. Its use for metabarcoding datas is sensibly the same and well documented. The deseq2_fun allows to process differential analysis as metacoder_fun, and users can choose the taxonomic rank, the factor to test and which condition to compare. DESeq2 algorithm uses negative binomial generalized linear models with VST normalization (Variance Stabilizing Transformation).

Main output is a list with:

- a plot showing Log2FoldChange value of each significant feature ( ${comparison}$plot).
- a table with statistics (LogFoldChange, pvalue, adjusted pvalue...) ( ${comparison}$table).

# DESeq2
deseq = deseq2_fun(data = data_filtered, output = "./analysis/07_all_res/DA/deseq/",
    column1 = "source_location", verbose = 1, rank = "Genus", comp = "milk^~^teat")

**MetagenomeSeq** MetagenomeSeq uses a normalization method able to control for biases in measurements across taxonomic features and a mixture model that implements a zero-inflated Gaussian distribution to account for varying depths of coverage. As deseq2_fun, metagenomeseq_fun returns a table with statistics and a plot with significant features for each comparison.

# MetagenomeSeq
mgSeq = metagenomeseq_fun(data = data_filtered, output = "./analysis/07_all_res/DA/metagenomeseq/",
    column1 = "source_location", verbose = 1, rank = "Genus", comp = "milk^~^teat")

**Figure 7.**
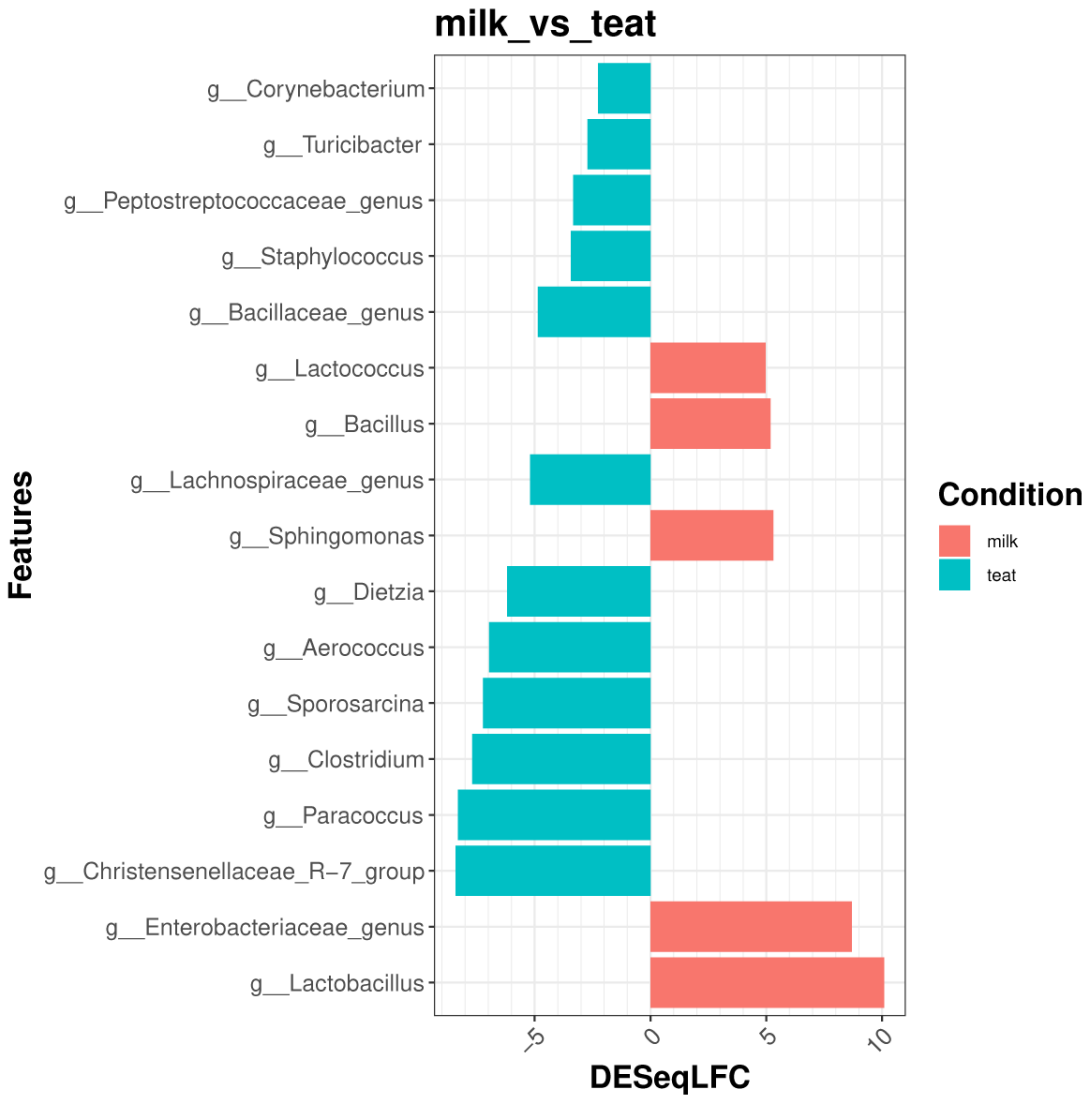
Top significant and differentially abundant genera between Milk and Teat samples.

**Results aggregation step** The aggregate_fun function allows to merge the results from the three differential analyses methods computed previously to obtain one unique table with all informations of significant differentially abundant features. Figure 7 shows the most significant and differential abundant Genera between the two environments.

# Aggregate results
resF = aggregate_fun(data = data_filtered,
    metacoder = "./analysis/07_all_res/DA/metacoder/metacoder_signif_Family.csv",
    deseq = "./analysis/07_all_res/DA/deseq/",
    mgseq = "./analysis/07_all_res/DA/metagenomeseq/",
    output = "./analysis/07_all_res/DA/aggregate_diff/",
    column1 = "source_location", column2 = NULL, verbose = 1,
    rank = "Genus", comp = "milk^~^teat")

The generated table include the following fields:

- seqid: ASV ID.
- Comparaison: Tested comparison.
- Deseq / metagenomeSeq / metacoder: differentially abundant with this method (0 no or 1 yes).
- sumMethods: sum of methods in which feature is significant.
- DESeqLFC: Log Fold Change value as calculated in DESeq2.
- absDESeqLFC: absolute value of Log Fold Change value as calculated in DESeq2.
- MeanRelAbcond1 / MeanRelAbcond2: Means relative abundance in condition 1 and 2.
- Condition: in which the mean feature relative abundance is higher.
- Taxonomy and representative sequence.

Here is an overview of the aggregate_fun table informing in which methods each feature is significant, their DESeq2 LogFoldChange value, taxonomy and representative sequences:

as.data.frame(head(apply(resF$table, 2, stringr::str_sub, 1, 10)))
#        seqid Comparaison DESeq metagenomeSeq metacoder sumMethods   DESeqLFC absDESeqLFC
# 1 a0bc0ee9c9  milk_vs_te     1             1         1          3  4.9664456   4.9664456
# 2 13b622bf4d  milk_vs_te     1             1         1          3  8.6935035   8.6935035
# 3 9c7d3af28a  milk_vs_te     1             1         1          3 -3.3346494   3.3346494
# 4 d47e07353a  milk_vs_te     1             0         1          2 -2.2647154   2.2647154
# 5 7f32bad90a  milk_vs_te     1             0         1          2 -2.7186798   2.7186798
# 6 ae592ee947  milk_vs_te     1             1         1          3  5.2998345   5.2998345
#   MeanRelAbcond1 MeanRelAbcond2 Condition     Domain  ...     Species   sequence
# 1     1.177130e-     2.996581e-      milk k__Bacteri  ...  s__Lactoco ATCTTCGGCA
# 2     9.035658e-     2.193079e-      milk k__Bacteri  ...  s__Enterob ATATTGCACA
# 3     1.310357e-     1.481454e-      teat k__Bacteri  ...  s__Peptost ATATTGCACA
# 4     1.510200e-     8.532074e-      teat k__Bacteri  ...  s__Coryneb ATATTGCACA
# 5     8.272207e-     7.662663e-      teat k__Bacteri  ...  s__Turicib ATCTTCGGCA
# 6     2.477752e-     5.291575e-      milk k__Bacteri  ...  s__Sphingo ATATTGGACA

### Miscellaneous function

**Figure 8.**
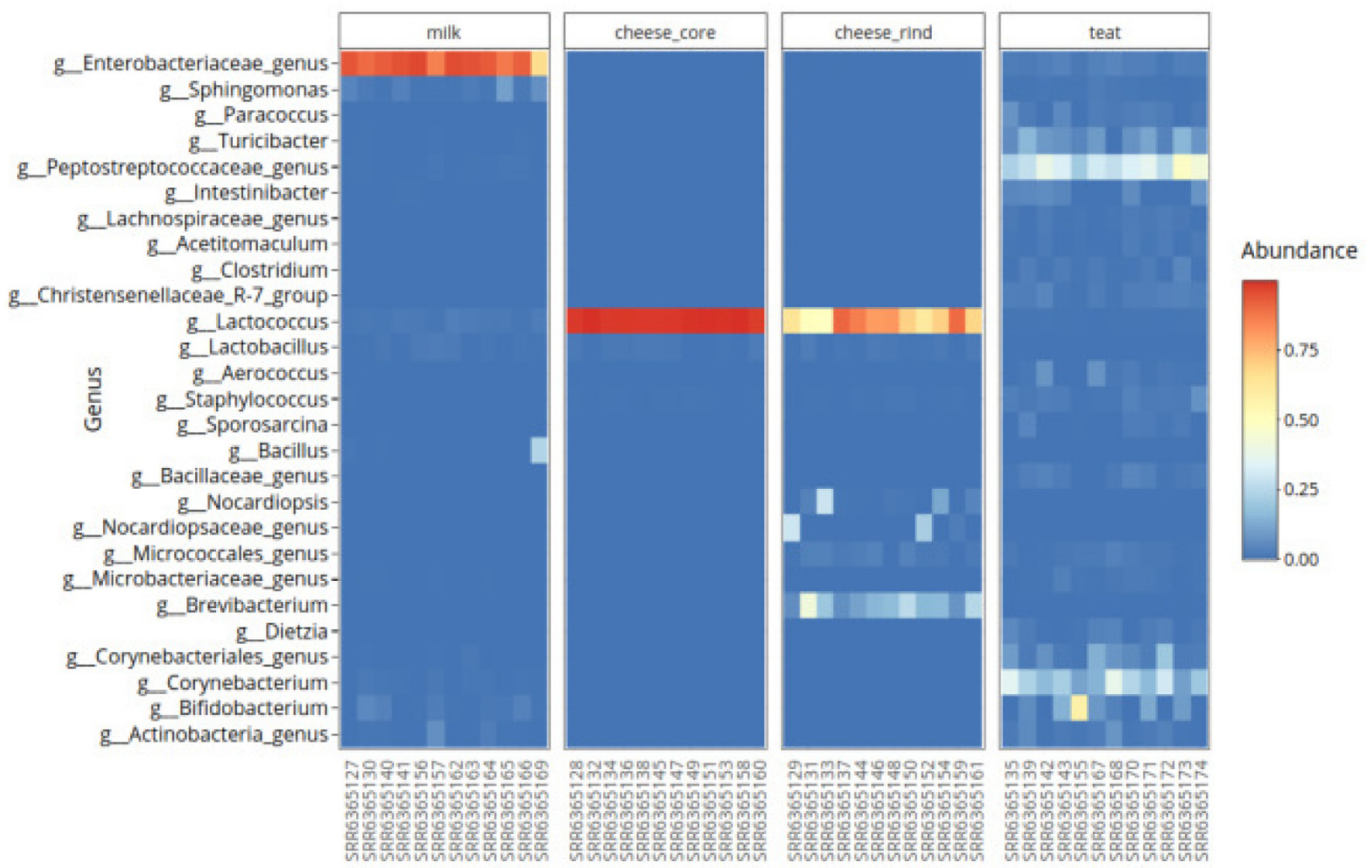
Heatmap of top genuses relative abundance.

***Heatmap*.** User can generate an interactive heatmap with heatmap_fun function to explore relative abundance of top taxa through samples as showed in Figure 8.

# heatmap
heatmap_plot = heatmap_fun(data = data_filtered, column1 = "source_location", top = 100,
     output = "./analysis/07_all_res/plot_heatmap/", rank = "Genus")

**Figure 9.**
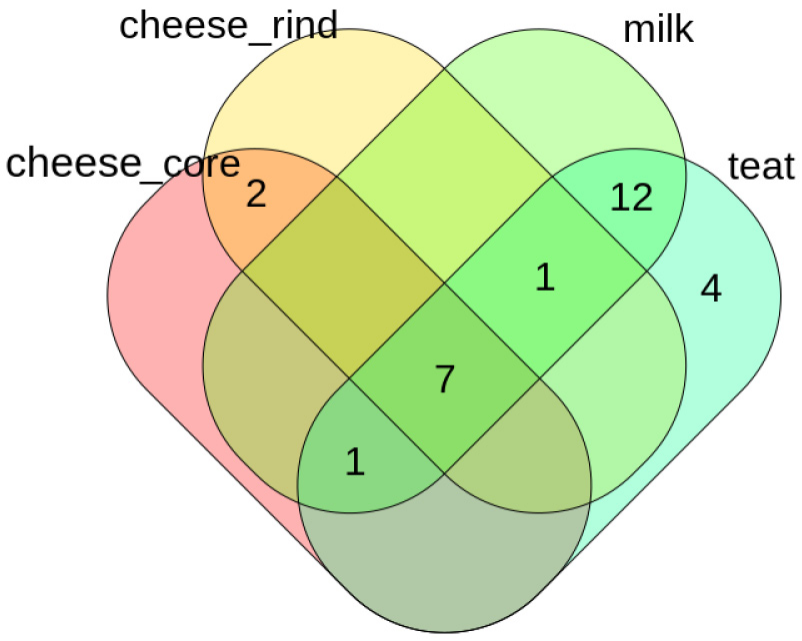
Venn plot of shared genuses.

***Venn diagram of shared taxa*.** Figure 9 shows Venn diagram comparing the three environment of this study, this method is useful to determine shared taxa between group of samples. This function uses venn::venn function which handles up to 7 groups comparison.

# venn
outvenn = ASVenn_fun(data = data_filtered, output = "./analysis/07_all_res/ASVenn/",
     rank = "Genus", column1 = "source_location", shared = TRUE)

ASVenn_fun generate a table allowing user to find which taxa are shared between conditions.

outvenn$TABf  #table with shared taxa, taxonomies and sequences

as.data.frame(head(apply(outvenn$TABf, 2, stringr::str_sub, 1, 10)))
#   teat milk cheese_rind cheese_core        ASV   taxonomy        seq
# 1    1    1           1           1 a0bc0ee9c9 k__Bacteri ATCTTCGGCA
# 2    1    1           1           1 13b622bf4d k__Bacteri ATATTGCACA
# 3    1    1           1           1 15875b7f67 k__Bacteri ATATTGCACA
# 4    0    0           1           1 fe8453c0a4 k__Bacteri ATATTGCACA
# 5    1    1           1           1 907e61bc24 k__Bacteri ATATTGCACA
# 6    0    0           1           1 fe1733ab7e k__Bacteri ATATTGCACA

### Export and compatibility

phy2cyto_fun allows to generate input files (SIF format) for Cytoscape which is useful to visualise shared taxa and easily modify each nodes and arrows position and aesthetic.

phy2tsv_fun function allows user to generate tabulated format abundance table at different taxonomic rank with following commnands:

for (i in c("ASV", "Species", "Genus", "Family")) {
     phy2tsv_fun(data_prune, output = glue::glue("./analysis/08_tsv_table/{i}_table/"),
          rank = i)
}

export_to_stamp_fun function creates two files that can be imported into the STAMP software.

export_to_stamp_fun(data = data, output = "./analysis/stamp/", correc = TRUE)

csv2phyloseq_fun function allows user to import data from other bioinformatic pipeline like FROGS, Qiime2. This function needs tabulated ASV, taxonomy, metadata and DNA sequence table as inputs. It can generate phylogenetic tree if missing and output a phyloseq object ready for downstream analyses.

## Conclusions

rANOMALY allows users to handle metagenomic data from raw sequences quality control to final differential analysis with ready to publish results in an easy and reproducible manner. Users have access to all sources of the rANOMALY package that can be deployed on any operating system or server allowing them to analyse anything from a few samples to several thousand. This workflow combines all of the latest developments in the field: the use of high resolution amplicon sequence variant, contaminant filtering, double automatic taxonomic assignation, integrated statistical analyses and four differential analyses with cross validation. rANOMALY help users those who don’t have big programming skills. And since rANOMALY uses phyloseq objects and standards, original functions from the phyloseq package can still be used to split, filter and select specific samples previously to be visualized and/or tested with rANOMALY functions. For exploratory analysis and interactive experience, a shiny application based on this workflow is under active development and will be shared with the community.

## Software availability

Up-to-date source code, and tutorials are available at: https://forgemia.inra.fr/umrf/ranomaly.

Package documentation is also provided at: https://umrf.pages.mia.inra.fr/ranomaly/use_case.html

Archived source code as at time of publication are available from: https://doi.org/10.5281/zenodo.4338833 ^26^

License: Creative Commons Attribution 4.0 International

Here we show all packages used in this workflow and their version numbers:

sessionInfo()
# R version 3.6.3 (2020-02-29)
# Platform: x86_64-pc-linux-gnu (64-bit)
# Running under: Ubuntu 18.04.5 LTS
#
# Matrix products: default
# BLAS:   /usr/lib/x86_64-linux-gnu/blas/libblas.so.3.7.1
# LAPACK: /usr/lib/x86_64-linux-gnu/lapack/liblapack.so.3.7.1
#
# locale:
#  [1] LC_CTYPE=fr_FR.UTF-8       LC_NUMERIC=C               LC_TIME=fr_FR.UTF-8        LC_COLLATE=fr_FR.UTF-8
#  [5] LC_MONETARY=fr_FR.UTF-8    LC_MESSAGES=fr_FR.UTF-8    LC_PAPER=fr_FR.UTF-8       LC_NAME=C
#  [9] LC_ADDRESS=C               LC_TELEPHONE=C             LC_MEASUREMENT=fr_FR.UTF-8 LC_IDENTIFICATION=C
#
# attached base packages:
# [1] stats     graphics  grDevices utils     datasets  methods   base
#
# other attached packages:
# [1] ranomaly_0.0.0.9000
#
# loaded via a namespace (and not attached):
#   [1] taxa_0.3.4                  tidyselect_1.1.0            htmlwidgets_1.5.1          RSQLite_2.2.0
#   [5] AnnotationDbi_1.48.0        grid_3.6.3                  BiocParallel_1.20.1        Rtsne_0.15
#   [9] devtools_2.3.2              IHW_1.14.0                  munsell_0.5.0              codetools_0.2-16
#  [13] withr_2.2.0                 colorspace_1.4-1            Biobase_2.46.0             phyloseq_1.30.0
#  [17] knitr_1.30                  rstudioapi_0.11             stats4_3.6.3               ggsignif_0.6.0
#  [21] slam_0.1-47                 GenomeInfoDbData_1.2.2      lpsymphony_1.14.0          hwriter_1.3.2
#  [25] bit64_0.9-7.1               rhdf5_2.30.1                rprojroot_1.3-2            vctrs_0.3.2
#  [29] generics_0.0.2              xfun_0.16                   lambda.r_1.2.4             R6_2.4.1
#  [33] GenomeInfoDb_1.22.1         locfit_1.5-9.4              bitops_1.0-6               microbiome_1.8.0
#  [37] DelayedArray_0.12.3         assertthat_0.2.1            scales_1.1.1               gtable_0.3.0
#  [41] processx_3.4.4              phangorn_2.5.5              rlang_0.4.7                genefilter_1.68.0
#  [45] splines_3.6.3               lazyeval_0.2.2              rstatix_0.6.0              broom_0.7.0
#  [49] reshape2_1.4.4              abind_1.4-5                 backports_1.1.8            tools_3.6.3
#  [53] usethis_1.6.3               ggplot2_3.3.2               ellipsis_0.3.1             gplots_3.1.0
#  [57] decontam_1.6.0              biomformat_1.14.0           RColorBrewer_1.1-2         BiocGenerics_0.32.0
#  [61] sessioninfo_1.1.1           Rcpp_1.0.5                  plyr_1.8.6                 zlibbioc_1.32.0
#  [65] psadd_0.1.3                 purrr_0.3.4                 RCurl_1.98-1.2             ps_1.4.0
#  [69] prettyunits_1.1.1           ggpubr_0.4.0                Wrench_1.4.0               viridis_0.5.1
#  [73] S4Vectors_0.24.4            SummarizedExperiment_1.16.1 haven_2.3.1                cluster_2.1.0
#  [77] fs_1.4.2                    DECIPHER_2.14.0             magrittr_1.5               RSpectra_0.16-0
#  [81] data.table_1.13.0           futile.options_1.0.1        pairwiseAdonis_0.0.1       openxlsx_4.1.5
#  [85] ranacapa_0.1.0              matrixStats_0.56.0          pkgload_1.1.0              evaluate_0.14
#  [89] hms_0.5.3                   xtable_1.8-4                XML_4.0-0                  VennDiagram_1.6.20
#  [93] rio_0.5.16                  jpeg_0.1-8.1                readxl_1.3.1               IRanges_2.20.2
#  [97] gridExtra_2.3               shape_1.4.4                 testthat_3.0.0             compiler_3.6.3
# [101] ellipse_0.4.2               tibble_3.0.3                KernSmooth_2.23-17         crayon_1.3.4
# [105] htmltools_0.5.0             venn_1.9                    corpcor_1.6.9              mgcv_1.8-33
# [109] tidyr_1.1.0                 geneplotter_1.64.0          RcppParallel_5.0.2         DBI_1.1.0
# [113] formatR_1.7                 MASS_7.3-53                 ShortRead_1.44.3           Matrix_1.2-18
# [117] ade4_1.7-15                 car_3.0-10                  permute_0.9-5              cli_2.0.2
# [121] quadprog_1.5-8              parallel_3.6.3              igraph_1.2.5               GenomicRanges_1.38.0
# [125] forcats_0.5.0               pkgconfig_2.0.3             GenomicAlignments_1.22.1   foreign_0.8-76
# [129] plotly_4.9.2.1              foreach_1.5.0               rARPACK_0.11-0             annotate_1.64.0
# [133] admisc_0.9                  multtest_2.42.0             XVector_0.26.0             stringr_1.4.0
# [137] callr_3.5.1                 digest_0.6.25               vegan_2.5-6                dada2_1.14.1
# [141] Biostrings_2.54.0           rmarkdown_2.5               cellranger_1.1.0           fastmatch_1.1-0
# [145] curl_4.3                    Rsamtools_2.2.3             gtools_3.8.2               lifecycle_0.2.0
# [149] nlme_3.1-149                jsonlite_1.7.1              Rhdf5lib_1.8.0             mixOmics_6.10.9
# [153] carData_3.0-4               futile.logger_1.4.3         desc_1.2.0                 viridisLite_0.3.0
# [157] limma_3.42.2                fansi_0.4.1                 pillar_1.4.6               metacoder_0.3.4
# [161] lattice_0.20-41             httr_1.4.2                  plotrix_3.7-8              pkgbuild_1.1.0
# [165] survival_3.1-12             glue_1.4.1                  remotes_2.2.0              zip_2.0.4
# [169] fdrtool_1.2.15              png_0.1-7                   iterators_1.0.12           glmnet_4.0-2
# [173] bit_1.1-15.2                stringi_1.4.6               metagenomeSeq_1.28.2       blob_1.2.1
# [177] DESeq2_1.29.13              latticeExtra_0.6-29         caTools_1.18.0             memoise_1.1.0
# [181] dplyr_1.0.0                 ape_5.4
