# Supplementary material for: rANOMALY: AmplicoN wOrkflow for Microbial community AnaLYsis
Source: F1000Res. 2021 Jan 7;10:7. [Version 1] doi: 10.12688/f1000research.27268.1 (PMC7836088; doi:10.12688/f1000research.27268.1)
Supplement: Peer review history

# Peer review: Reviewer response for version 1

**DOI:** 10.5256/f1000research.30130.r76936

**Published:** 2021-01-22

**Authors:** Simon Roux

**Affiliations:** 1 Department of Energy Joint Genome Institute, Berkeley, CA, USA

This manuscript describes an R pipeline (rANOMALY) that connects together in a single simplified R workflow many of the standard tools now routinely used in metabarcoding data analysis. I have no doubt that such an integrated pipeline will be very useful for many researchers, especially ones not experts in bioinformatics. The modular design is also well suited for future changes and evolution of the pipeline.

 The manuscript itself is very clear, nicely outlines how the different components of the pipeline are connected, and also clearly distinguish between which functions are provided by this pipeline and which functions are provided by third-party tools or packages. My only major comment is about user input (either user questions or bug reports), for which there is currently no process or web page mentioned in the manuscript. For instance, should users report bugs in the gitlab repo ( https://forgemia.inra.fr/umrf/ranomaly/-/issues)? (Incidentally, I did encounter an unexpected error with DeSeq: Error in `assays<-`(`*tmp*`, value = `*vtmp*`) :

 please use 'assay(x, withDimnames=FALSE)) <- value' or 'assays(x, withDimnames=FALSE)) <- value' when the dimnames on the supplied assay(s) are not identical to the dimnames on DESeqDataSet object 'x' In addition: Warning message: In DESeqDataSet(se, design = design, ignoreRank) :

 some variables in design formula are characters, converting to factors).

 If the authors do intend users to submit gitlab issues, this should be specified in the conclusion and/or the software availability sections. If not, the alternative(s) path to provide feedback and report issues should be clearly described.

 Other than that, I only have minor comments on the manuscript which are listed below:

- Abstract:
  “uses amplicon sequence variants (ASV) level for”. Shouldn’t this read “uses amplicon sequence variants (ASV) for”?
  In the keywords, I would argue “metagenomics” should be “metabarcoding”
- p. 3 Introduction:
  “Studies of microbial communities tends to” should be “Studies of microbial communities tend to”
  “(FROGS ^1^ , qiime ^2^ )”: it should be made clear that these are examples of softwares but many more exist, e.g. by changing to “(e.g. FROGS ^1^, qiime ^2^)”, or explicitly stating “such as FROGS ^1^ or qiime ^2^”.
  “allowing to easy implementation of” should be “allowing for easy implementation of” or “allowing to easily implement”.
- p. 3 Raw sequence processing:
  “This improves” should be clarified as “This approach improves” or “This tool improves” (to make it obvious that “this” refers to dada2).
  “an additional step of cutadapt” should be “an additional step using cutadapt”.
- p. 4 Taxonomic assignment:
  “with representing sequences” should probably be clarified as “with representative sequences for each ASV” (since the concept of “representative sequences” has not been described earlier)
  “IDTAXA formatted” should be “IDTAXA-formatted”
- p. 4 Phylogenetic tree:
  “with AlignSeqs function from DECIPHER package” should be “with the AlignSeq function from the DECIPHER package”
  “computes the likelihood of the phylogenetic tree” This may suggest that a tree exists and that this function evaluates the likelihood of this existing tree, but I believe the first function actually builds a tree? So there may be a clause missing here, e.g. “And finally, the neighbour joining function is used to build a phylogenetic tree based on the distance matrix, and the pml function computes the likelihood of the phylogenetic tree.”?
- p. 5 Install:
  The authors may want to add a “library(devtools)” line after the install of the package devtools and before installing the package ranomaly, in case some users already have the devtools package installed.
  The authors should also specify that a user will need to replace “PATHTOWORKINGDIRECTORY” with the path to their working directory on their own computer. (The same is true in the Taxonomic assignment section when users have to specify a path to the taxonomic databases).
- p. 6 Processing of raw sequences:
  “files name match samples name” should be “file names match sample names”
- p. 6 Taxonomic assignment:
  “givenby” should be “given by”
  “different genus” should be “different genera”
  “correction step” should be removed, or clarified as e.g. “as part of a correction step”
  “your own” should be “their own”
- p. 8 Rarefaction curves:
  On my version of R, I have to call “rareplot” after the line “rareplot = rarefaction(data_filtered, "source_location", 100)” otherwise no plot appears. If this is the case in most versions (which I believe it is), this line (“rareplot”) should be included in the authors’ instructions so that a user would not be confused to not see any plot after calling the line “rareplot = rarefaction(data_filtered, "source_location", 100)” (this is also true for the other interactive plots, e.g. community composition, alpha and beta diversity, etc).
- p. 8 Community composition plot:
  "source_ location": there seems to be an extra space between “source_” and “location” here
- p. 10 Beta diversity:
  “funtion” should be “function”
  “on matrix distance” should be “on the distance matrix”
  “A pairwise-PERMANOVA is processed” should be “A pairwise-PERMANOVA is performed”
- p. 11 Differential analysis:
  “of the use of multiple” would be more clear as “of using multiple”
  “cross validate” should be “cross-validate”
  “which factor to the test” should be “which factor to test” or “which factor to perform the test on”
  “which group this taxa is more abundant” should be “which group this taxa is more abundant in”
- p. 12 DESeq2:
  “and well documented”: Could the authors add 1 or 2 references here?
- p. 15 Venn diagram:
  Shouldn’t “the three environment” be “the four environments”.
- p. 15 Export:
  “function allows user” should be “function allows users” (also for csv2phyloseq_fun).
- p. 15 Conclusion:
  “metagenomic data” should be “metabarcoding data”
  “help users those” should be “help users”
  “big programming skills” should be “advanced programming skills”
  “previously to be visualized and/or tested” should be “prior to visualization/test” or “before being visualized and/or tested”

Are the conclusions about the tool and its performance adequately supported by the findings presented in the article?

Yes

Is the rationale for developing the new software tool clearly explained?

Yes

Is the description of the software tool technically sound?

Yes

Are sufficient details of the code, methods and analysis (if applicable) provided to allow replication of the software development and its use by others?

Yes

Is sufficient information provided to allow interpretation of the expected output datasets and any results generated using the tool?

Yes

Reviewer Expertise:

metagenomics, microbial ecology, phage genomics, viral ecology

I confirm that I have read this submission and believe that I have an appropriate level of expertise to confirm that it is of an acceptable scientific standard.

# Peer review: Reviewer response for version 1

**DOI:** 10.5256/f1000research.30130.r76934

**Published:** 2021-01-12

**Authors:** Florent Murat

**Affiliations:** 1 Center for Molecular Biology of Heidelberg University (ZMBH), Heidelberg, Germany

Theil and Rifa developed here the R package rANOMALY for data analysis of 16S and ITS amplicons based sequencing. They provide a workflow and a toolkit that are very useful for bioinformaticians as well as for beginners who want to perform complete marker gene sequencing data analyses from fastq treatment (contaminant filtering, taxonomic assignment, alpha and beta diversity indexes) to statistical analysis (using three main differential analysis methods, DESeq2, Metacoder, metagenomeSeq) in an automatic, step by step, and reproducible way. Thus, this is a suitable workflow for exploratory and educational purposes. I was pleased to be able to reproduce all analyses and figures following the use case of the paper.

 Hence, I only have a few minor issues that should be discussed:

1. The authors mentioned that the developed package is customizable. This is an important aspect given that projects are different from one to the other, and bioinformatic tools evolve tremendously fast so it will be crucial to adapt/update the workflow accordingly. The workflow actually allows this, however, it would be useful (particularly for beginners) to provide a vignette/tutorial on the gitlab page (and/or in a section of the paper) showing how this can be done with simple examples. The use case is based on Illumina sequences, a use case dealing with Ion torrent sequences could be relevant in this context.
2. The use case is based on a dataset from Fretin *et al.* 2018, it would be relevant to interpret the results of the workflow, and to describe to what extent they match or complement the results of the previous analysis. This would be relevant to benchmark the workflow and highlight the novelties brought by this analysis.
3. The access to the use case fastq sequences and to the IDTAXA formatted databases is given via links in the text. It would be convenient for users working with remote servers to download it with command lines. Please provide command lines to do so (via wget for instance), either in the text or on the gitlab page.
4. There is a typo on page 6, the IDTAXA formatted database that can be downloaded from the link is DAIRYdb_v1.2.4_20200603_IDTAXA.rdata and not DAIRYdb_v1.2.4_20200604_IDTAXA.rdata.
5. Page 8: In the “bars_fun" function, additional space has been introduced between “source_” and “location” leading to an error if one runs this piece of code.
6. Page 9: In the “diversity_alpha_fun” function, the font has been modified for the closing double quotes after “Observed” and “Shannon”. Thus, this leads to an error if one runs this piece of code.
7. Page 15: In the “phy2tsv" function, the phyloseq object “data_prune" was never created upstream. Would it be better to test the function “phy2tsv” with the phyloseq object “data” previously created in the use case?

Are the conclusions about the tool and its performance adequately supported by the findings presented in the article?

Yes

Is the rationale for developing the new software tool clearly explained?

Yes

Is the description of the software tool technically sound?

Yes

Are sufficient details of the code, methods and analysis (if applicable) provided to allow replication of the software development and its use by others?

Yes

Is sufficient information provided to allow interpretation of the expected output datasets and any results generated using the tool?

Yes

Reviewer Expertise:

Genomics, Bioinformatics, Evolution

I confirm that I have read this submission and believe that I have an appropriate level of expertise to confirm that it is of an acceptable scientific standard.
